# The Potential for iPS-Derived Stem Cells as a Therapeutic Strategy for Spinal Cord Injury: Opportunities and Challenges

**DOI:** 10.3390/jcm4010037

**Published:** 2014-12-29

**Authors:** Mohamad Khazaei, Ahad M. Siddiqui, Michael G. Fehlings

**Affiliations:** 1 Department of Genetics and Development, Toronto Western Research Institute, University Health Network, Toronto, M5T 2S8, ON, Canada; E-Mails: mohamad.r.khazaei@gmail.com (M.K.); ahadmsiddiqui@gmail.com (A.M.S.); 2 Department of Surgery, University of Toronto, Toronto, M5T 1P5, ON, Canada; 3 Institute of Medical Sciences, University of Toronto, Toronto, M5S 1A8, ON, Canada

**Keywords:** spinal cord injury, clinical translation, neuroprotection, cell therapy, neuroregeneration

## Abstract

Spinal cord injury (SCI) is a devastating trauma causing long-lasting disability. Although advances have occurred in the last decade in the medical, surgical and rehabilitative treatments of SCI, the therapeutic approaches are still not ideal. The use of cell transplantation as a therapeutic strategy for the treatment of SCI is promising, particularly since it can target cell replacement, neuroprotection and regeneration. Cell therapies for treating SCI are limited due to several translational roadblocks, including ethical and practical concerns regarding cell sources. The use of iPSCs has been particularly attractive, since they avoid the ethical and moral concerns that surround other stem cells. Furthermore, various cell types with potential for application in the treatment of SCI can be created from autologous sources using iPSCs. For applications in SCI, the iPSCs can be differentiated into neural precursor cells, neurons, oligodendrocytes, astrocytes, neural crest cells and mesenchymal stromal cells that can act by replacing lost cells or providing environmental support. Some methods, such as direct reprogramming, are being investigated to reduce tumorigenicity and improve reprogramming efficiencies, which have been some of the issues surrounding the use of iPSCs clinically to date. Recently, iPSCs have entered clinical trials for use in age-related macular degeneration, further supporting their promise for translation in other conditions, including SCI.

## 1. Current Outlook on the Pathophysiology and Treatment of Spinal Cord Injury

### 1.1. Epidemiology of Spinal Cord Injury

Dislocation or fracture of the spine in the neck or back as a result of vehicle accidents, falls, sports accidents, work accidents or other causes commonly results in spinal cord injury (SCI). The seriousness of the damage varies depending on the severity of the injury and the level of injury. Over half of SCIs occur at the cervical level of the spinal cord [1]. The global prevalence of SCI varies between 250 and 906 per million of the population depending on global region [1,2,3]. There have been many advances in the medical, surgical and rehabilitative treatment of SCI in the last few decades; however, these treatments result in limited functional recovery after injury.

### 1.2. Pathophysiology of SCI

The mechanical crushing, stretching or rupture of the spinal cord at the time of injury leads to axonal damage, quick necrotic death and loss of neurons and glia, which are collectively referred to as the primary injury [4,5,6]. Axon damage and disruption of the cell membrane that occurs during the primary injury results in a cascade of molecular and signaling pathways that initiate a series of secondary injuries to the spinal cord. Formation of free radicals and oxidative stress as a consequence of secondary injuries result in more neuronal and glial death, mainly due to apoptosis [7]. Disintegration of myelin and demyelination are another consequence of secondary injury in the spinal cord. The mechanical insult to the spinal cord also results in the disruption of the blood spinal cord barrier (BSCB). This increases the permeability of the BSCB, allowing the infiltration of immune cells from the blood and increasing inflammation, which augment secondary injury [8]. The activation of astrocytes results in reactive gliosis and subsequent formation of the glial scar which acts as a physical and chemical barrier that inhibits axon regeneration. Progressive loss of neurons and glial cells results in the formation of a cystic cavity in the spinal cord [5,9,10].

### 1.3. Approaches and Progresses towards the Treatment of SCI

The current treatment options for SCI are mainly focused on stabilizing the spine, preventing the progress of secondary injuries and controlling inflammation. Fractured vertebrae and bone fragments that compress the spinal cord may need to be surgically removed by spinal decompression surgery [11]. Corticosteroid drugs (like methylprednisolone) may be used within 8 h of the injury, although their application is controversial. Methylprednisolone appears to work by modulating inflammation near the site of injury and reducing damage to nerve cells [12]. After the initial treatment and stabilization of patients with an SCI, much of the current treatment approaches are geared toward rehabilitation. However, there are many promising advancements in research towards protecting surviving neural cells from further damage, stimulating axonal regeneration and replacing damaged nerve or glial cells. Several medications that can increase neuronal survival and reduce inflammation are in clinical trials, including corticosteroids, minocycline, erythropoietin and gangliosides [12]. Riluzole is a drug with neuroprotective effects that has been investigated by our laboratory as a part of an international, multicenter effort sponsored by AOSpine and the North American Clinical Trial Network [13]. Therapeutic interventions to promote axonal regeneration have also entered clinical trials. In a phase I/IIa clinical trial, in which our laboratory was also involved, the RhoA inhibitor C3 transferase (Cethrin) was tested on patients with SCI. The observed motor recovery in this open-label trial suggests that inactivation of RhoA may increase neurological recovery after complete SCI [14,15].

### 1.4. Cell Therapy: Promise and Progress

Stem cell transplantation is a promising therapeutic strategy for the treatment of SCI that works through several different mechanisms [16,17]. Preclinical studies have shown encouraging beneficial effects of cell therapies in animal models of SCI. Cell therapies have been shown to have their therapeutic effect through many mechanisms that target different events occurring during the primary and secondary phases of SCI. One of these mechanisms is the replacement of cells that are lost or damaged during the injury, through differentiation or transdifferentiation into mature neurons and through myelination of oligodendrocytes. Some transplanted cells render their therapeutic effect by providing neurotrophic factors that are crucial in order to enhance neuronal regeneration and survival. Some other cell types are beneficial to SCI through downregulation of inhibitory molecules, immunomodulation, modulation of the environment and extracellular matrix or by providing scaffold support for the regeneration of axons [16,17,18].

Several differentiated, multipotent or pluripotent cell types have been investigated so far for the treatment of SCI. Some of these cells have entered clinical trials. One such study is a phase I/II trial using human neural progenitor cells (NPCs) sponsored by Stem Cells Inc. (Newark, CA, United States of America). Our centre (Toronto Western Hospital) is involved in this trial, in collaboration with other centers at the University of Calgary and the University of Zurich. The first patient in this trial was treated in Toronto in February, 2014. Despite these advances, stem cell therapy for SCI is limited by the availability of the ideal cell source, the control and safety of the transplantation and the ethical and logistical challenges surrounding the use of stem cells.

Here, we briefly describe some of the most important cell types that have been investigated so far for the treatment of SCI. For a more thorough review on the application of these cells, refer to the recent review from our laboratory on this topic [17].

#### 1.4.1. Neural Progenitor Cells

Neural progenitor cells (NPCs) have attracted great interest as a potential source for replacing damaged or lost neurons and glia in SCI [16]. Our laboratory and others have shown that transplantation of rodent and human NPCs into the spinal cord improves neural repair and regeneration, as well as functional recovery following traumatic SCI in rodents. This occurs via cell replacement and plasticity, remyelination and nutrient secretion, increasing axonal regeneration and immunomodulatory effects [19,20,21,22,23,24]. Although adult NPCs, derived from the CNS, are attractive for use after SCI due to their neural commitment and lack of tumorigenicity [20,22,24], the derivation of adult or embryonic NPCs for autologous transplantation is not feasible. This is due to the fact that these cells are collected from the brains of aborted fetuses or post-mortem patients, which possibly excludes their application in the clinical treatment of SCI. Furthermore, concerns regarding donor cell rejection have been problematic in SCI, in which activated inflammatory responses can present an intrinsically hostile environment to any allogeneic grafts.

#### 1.4.2. Mesenchymal Stromal Cells

Mesenchymal stromal cells (MSCs) are multipotent cells that originate from the mesodermal germ layer. Several labs have studied the effect of MSCs for the treatment of SCI. These studies demonstrate that MSCs exert their beneficial effect mostly by providing immunomodulation, trophic support, environmental modification and by providing physical scaffolding for elongating axons [25,26,27,28], resulting in improvement of locomotor function [29,30,31,32,33]. Due to poor engraftment and limited differentiation under *in vivo* conditions, MSCs do not have the potential to be used for cell replacement therapy for SCI, and their therapeutic effect is limited to providing trophic support. An additional limitation is the potential of MSCs to differentiate into unwanted mesenchymal lineages.

#### 1.4.3. Schwann Cells

Schwann cells (SCs) are one of the first cell types to have been used for the treatment of SCI. In the past two decades, many studies have demonstrated positive results and potential for SC transplantation as a therapy for SCI. They may do this by sustaining regeneration and through remyelination of damaged CNS axons, as well as by secreting several neurotrophic factors (such as NGF, BDNF and CNTF) [34] that aid the survival and intrinsic regeneration ability of damaged neurons. SCs have also been investigated in a clinical trial for the treatment of SCI [35]. In this trial, SCs were transplanted into the spinal cord one year after injury. This study demonstrated no adverse effects from SC transplantation, and one patient showed improvements in motor and sensory functions combined with extensive rehabilitation [35].

#### 1.4.4. Olfactory Ensheathing Glia

Olfactory ensheathing glia (OEG) are a type of myelinating cell derived from the olfactory mucosa. Like SCs, OEGs have also been transplanted as myelinating cells for the treatment of SCI in numerous studies in animal models of SCI. OEGs have been shown to facilitate remyelination and tissue scaffolding and can stimulate the regeneration of lesioned axons [36,37]. OEGs have also entered into clinical trials for the treatment of SCI. In one trial, no complications were reported one year after transplantation of OEG, but no functional recovery on the ASIA (American Spinal Injury Association) scale was found [38,39].

#### 1.4.5. Embryonic Stem Cell-Derived Cells

The isolation and propagation of the various cells types discussed above is difficult, and it is often a tedious and lengthy process to produce sufficient cells for treatment of SCI. The optimal time point for the application of cell therapy for SCI patients is 2–4 weeks after the injury [22,40], and it is important to have a sufficient amount of cells at this time window ready for transplantation. Embryonic stem cells (ESCs) are pluripotent cells derived from the inner cell mass of blastocysts with the ability to replicate indefinitely and the potential to differentiate into the cell types discussed above and, thus, may be useful as an accessible source for providing these cells for SCI treatment. Several studies have shown the beneficial effects of cells derived from ESCs in functional recovery in animal models of SCI [41,42,43,44,45,46]. Although providing a sufficient quantity of multipotent cells and differentiated ESCs is more feasible and requires less time, there are ethical issues concerning the destruction of human embryos or fertilized oocytes to obtain such stem cells. This has been a major impediment to developing clinically useful stem cell sources and to using them in clinical applications. Furthermore, there is the possibility of tumorigenesis due to incomplete differentiation.

## 2. Induced Pluripotent Stem Cells

The discovery of induced pluripotent stem cells (iPSCs) by Takahashi and Yamanaka in 2006 [47] opened novel opportunities in providing pluripotent stem cells for the treatment of patients with SCI and other injuries/diseases. They showed that stem cells with properties similar to ESCs could be generated from mouse fibroblasts by simultaneously introducing four factors: Oct4, Sox2, Klf2 and c-Myc [47]. In 2007, they reported that a similar approach was applicable for human fibroblasts to generate human iPSCs [48]. At the same time, James Thomson’s group also reported the generation of human iPSCs using a different combination of factors including: Oct4, Sox2, Nanog and Lin28 [49]. Since iPSCs can be derived directly from adult tissues, they can be made in a patient-specific manner that circumvents ethical and moral concerns while allowing for autologous transplantation.

### 2.1. Methods of Generating iPSCs

It is very important to have a safe and reliable method for the generation of iPSCs for clinical purposes. To reprogram the somatic cells into a pluripotent state, reprogramming factors should be introduced to the cells. Different combinations of reprogramming factors can be used with different efficiency and outcomes [50,51], which may be critical for clinical applications. Reprogramming methods that do not use the oncogene, c-Myc, are desirable to reduce the risk of tumor formation, but methods that exclude c-Myc are associated with significantly lower reprogramming efficiency [52]. Recently, the Yamanaka group has shown that the transcription factor, Glis1, can be used as a substitute for c-Myc for the induction of pluripotency [53].

The suitability of iPSCs for use in the clinic is also dependent on the method by which the reprogramming factors are delivered to the cell. Traditionally, lentiviruses have been used to deliver the reprogramming factors, but random integration of the lentivirus DNA into the host genome raises concerns about the risk of tumorigenicity and the safety of this method. Other viruses, like adenovirus [54] and Sendai virus (SeV) [55], have also been used as less risky options. Adenovirus is considered to be safer than lentivirus, because it does not incorporate any of its own genes into the targeted host and, thus, avoids the potential for insertional mutagenesis [54]. SeV has higher efficiency in infecting a wide spectrum of host cell species and tissues compared to adenoviruses. Furthermore, SeV vectors replicate in the form of negative-sense single-stranded RNA in the cytoplasm of infected cells, which do not go through a DNA phase nor integrate into the host genome [55].

Various studies have recently described the induction of pluripotency without the use of viruses, but instead by using recombinant proteins [56], mRNAs [57], microRNAs [58], episomal vectors [59] and even removable transposons [60]. The piggyBac transposons have been shown to be able to deliver the reprogramming factors without leaving any footprint mutations in the host cell genome. The piggyBac system involves the re-excision of exogenous genes, which eliminates issues, such as insertional mutagenesis [61].

Another exciting approach that has been investigated recently is the use of small molecules and chemical compounds that can mimic the effects of transcription factors. The histone deacetylase (HDAC) inhibitor, valproic acid, has been shown to be able to mimic the signaling that is caused by the transcription factor, c-Myc, and can be used instead of c-Myc for reprogramming [62]. A similar type of compensatory mechanism has been proposed to mimic the effects of Sox2 by inhibition of histone methyl transferase (HMT) with BIX-01294 in combination with the activation of calcium channels in the plasma membrane [63]. More recently, Deng *et al.* (2013) showed that iPSCs could be created without any genetic modification. They used a cocktail of seven small-molecule compounds, including DZNep (3-deazaneplanocin A), to induce mouse somatic cells into stem cells, which they called CiPS (chemically induced pluripotent stem) cells, with an efficiency of 0.2%, comparable to those using standard iPSC production techniques [64].

### 2.2. Cell Sources for Generating iPSCs

**Figure 1:**
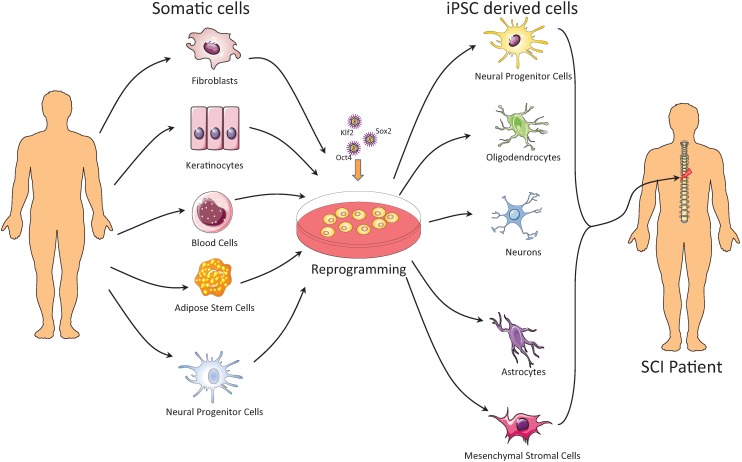
Several different types of cells have been used to produce iPSCs, including fibroblasts, neural progenitor cells, keratinocytes, melanocytes, CD34^+^ cells, cord blood cells and adipose stem cells. The next step, after generating iPSCs, towards the treatment of spinal cord injury (SCI) is to differentiate the iPSCs to the appropriate multipotent or differentiated cell type that can be used for the treatment of SCI. To date, several different cell types have been successfully derived from iPSCs and have been transplanted into SCI animal models, including neuronal progenitor cells, neurons, oligodendrocytes, astrocytes, neural crest cells and mesenchymal stromal cells.

Along with choosing the right reprogramming factors and delivery method for the generation of iPSCs, it is also important to use a source of cells that will generate the desirable cell types for transplantation into the spinal cord. Several different types of cells have been used to produce iPSCs, including fibroblasts, neural progenitor cells, keratinocytes, melanocytes, CD34^+^ cells, hepatocytes, cord blood cells and adipose stem cells (Figure 1).

**Figure 2:**
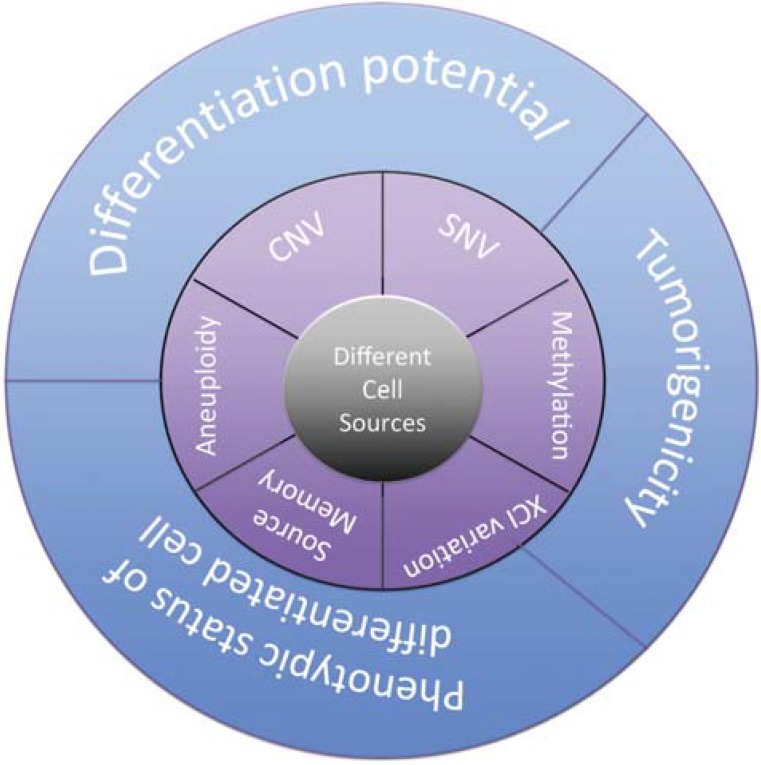
The epigenetic and genetic variations among different cell sources used for reprogramming can affect the properties of the iPSC and its differentiation to the target cell type. The characteristics of the different cell sources are at the core of defining how easily hiPSCs (human induced pluripotent stem cells) can be produced and their characteristics. Variations in aneuploidy, subchromosomal copy number variations (CNV), single-nucleotide variations (SNV), methylation, X chromosome inactivation (XCI) and source memory in the different cell sources can affect the differentiation potential, tumorigenicity and phenotypic status of hiPSCs. Aneuploidy is an abnormality in chromosome number. It is estimated that 13% of hiPSC cultures have karyotype abnormalities, commonly trisomy 12 [68]. CNVs are alterations in the DNA that lead to an abnormal number of a segment of the chromosome. They can occur around pluripotency genes, such as NANOG on chromosome 12 [67]. There can be as many as a dozen SNVs in a hiPSC line that may be inherited from their somatic cell source [69]. XCI is a process where one of the X chromosomes in the cell isolated from a female source is inactivated, so that it does not receive a double dose of the gene product. Reprogramming of certain cells may be more prone to XCI variations than others. Different lines of hiPSCs may have different methylation states, as shown by the fact that blood-derived iPSCs have an enhanced ability to become blood cells. This type of source memory is important to consider, since iPSCs can dedifferentiate after reprogramming to the source cell type [78].

iPSCs can even be derived from terminally-differentiated post-mitotic neurons. The cell types that are reprogrammed to become iPSCs can influence the differentiation capacity of the resultant iPSCs, due to epigenetic memory and genetic variations of their original cell line (Figure 2) [65,66,67,68,69]. The ideal iPSC source for the treatment of SCI should reprogram efficiently, be able to be isolated in large quantities in a reasonable period of time and, more importantly, should be able to differentiate into the desired multipotent/differentiated cell types that are required for the treatment of SCI. The efficiency of cell reprogramming varies among different cell types. For example, human keratinocytes from skin biopsies can be reprogrammed to pluripotency at a much higher frequency and more quickly than fibroblasts [70]. On the other hand, the iPSCs derived from keratinocytes have an increased tendency to differentiate into NPCs than do iPSCs from CD34^+^ blood cells [66]. More research is needed to determine the best starting somatic cell for iPSC generation that allows for reproducible differentiation into NPCs and other multipotent cell types for transplantation into SCI.

#### 2.2.1. Skin Fibroblasts

Skin fibroblasts are one of the most used cell types for reprogramming. Adult human fibroblasts can be easily isolated and maintained in culture [71,72], which makes them ideal for autologous transplantation in SCI patients. However, it takes a long time to reprogram these cells into iPSCs. Three to four weeks are required for expanding fibroblasts taken from human skin biopsy [73]. It takes another three to four weeks for iPSC colonies to appear [73]. Even after two months of culturing, the reprogramming efficiency of adult human fibroblasts from the skin is only 0.01% when the four Yamanaka factors are used and can be even lower if three or less of the factors are used [62]. Yamanaka postulated that since fibroblasts are terminally-differentiated cells, they require greater energy to reprogram than cells that are less differentiated [74]. However, recent studies have shown that it is possible to enhance the efficiency of iPSC generation by up to 100-fold, by using different combinations of reprogramming factors [51].

#### 2.2.2. Keratinocytes

There has been some interest in using keratinocytes for reprogramming, because they can be easily obtained from the human foreskin with minimal invasiveness [75]. This presents the opportunity to use keratinocyte-derived iPSCs for autologous transplantation in patients with SCI. Keratinocytes take longer to expand than fibroblasts, but can be reprogrammed more quickly (10 days) and have a higher reprogramming efficiency [76]. The higher reprogramming efficiency may be due to the higher levels of endogenous Klf4 and C-Myc [76], meaning that these cells require less energy to reach pluripotency. Although it is thought that cells isolated from younger sources are better suited for reprogramming, Linta *et al.* were able to achieve a reprogramming efficiency of 2.8% using adult keratinocytes transfected with the Yamanaka factors using lentiviruses [77]. This demonstrates the importance of considering the appropriate age and reprogramming conditions, as well as the cell type when determining the ideal method for developing iPSCs.

#### 2.2.3. Melanocytes

Like fibroblasts, melanocytes can also be isolated from skin biopsies. Melanocytes contain high levels of endogenous Sox2, so they can be reprogrammed using just the other three factors [79]. In addition, melanocytes take only 10 days to reprogram and have been shown to have a reprogramming efficiency of 0.19% [79]. All of this suggests that melanocytes may be a better option for use as sources for iPSCs than fibroblasts for autologous transplantation.

#### 2.2.4. CD34^+^ Cells

CD34^+^ cells isolated from human umbilical cord blood and peripheral blood have been used to generate iPSCs [80]. The collection of CD34^+^ cells from the peripheral blood of SCI patients is not thought to be ideal, since it has to be collected from patients undergoing granulocyte colony stimulating factor (G-CSF) mobilization [80]. G-CSF use is associated with increased risk of complications and side effects [81], which might not be well tolerated by SCI patients. CD34^+^ cells have a low reprogramming efficiency of 0.01%–0.02% with the Yamanaka factors [80]. The reprogramming efficiency is even lower for CD34^+^ cells isolated from umbilical cord blood [82].

#### 2.2.5. Cord Blood Cells

Umbilical cord blood may be a better source of iPSCs, since the method of isolation is less invasive, and they can be cryopreserved for more than five years and still be used to generate iPSCs [83]. Another benefit of cord blood is that many cord blood banks exist worldwide. CD133^+^ cells from umbilical cord blood can be reprogrammed to iPSCs using just Oct4 and Sox2 with a reprogramming efficiency of 0.45% [83]. Endothelial cells can also be isolated from cord blood and reprogrammed to iPSCs [84]. Cell isolated from the umbilical cord have primitive characteristics that make them ideal for reprogramming, since they may be epigenetically closer to iPSCs than other differentiated cells [85]. However, iPSCs derived from the umbilical cord cannot be considered as sources of autologous transplantation for SCI patients, unless the patient had already deposited his/her umbilical cord in cord blood banks after birth.

#### 2.2.6. Adipose Stem Cells

Adipose stem cells are multipotent cells that are collected by lipoaspiration [86]. As many as 100 million cells can be isolated from a 300-mL sample and can be expanded for reprogramming in approximately 48 h [87]. Using the Yamanaka factors, adipose stem cells can be reprogrammed in 10 to 15 days at a reprogramming efficiency of 0.2% [87]. Adipose stem cells express high levels of Klf4, and their multipotent nature would theoretically make them require fewer epigenetic changes to reach pluripotency [88].

#### 2.2.7. Neural Progenitor Cells

Multipotent cells, such as neural progenitor cells (NPCs), require fewer factors to be reprogrammed. Human fetal NPCs can be reprogrammed in seven to eight weeks using only Oct4 [89]. However, the reprogramming efficiency is very low, at 0.004%. NPCs are not an ideal source for generating iPSCs for use in the treatment of SCI, because they are not readily available and there are ethical issues associated with their use.

## 3. Using iPSC-Derived Cells for Treatment of SCI

The next step towards the treatment of SCI after generating iPSCs is to differentiate the iPSCs into the appropriate multipotent or differentiated cell type that can be used for the treatment of SCI (Figure 1). Due to the high risk of teratoma formation, the differentiation process should be “definitively” completed, and the cell population should be devoid of pluripotent cells. The direct injection of iPSCs into the injured spinal cord can be problematic. In an un-published study, Hodgetts *et al.* transplanted undifferentiated hiPSCs into the spinal cord in a thoracic level 7 (T7) contusion model of SCI in nude rats at seven days post injury [90]. They did not observe any significant improvement in motor function by five weeks after SCI with this method, despite subtle differences in some neuronal marker expressions at the lesion site [90].

**Figure 3:**
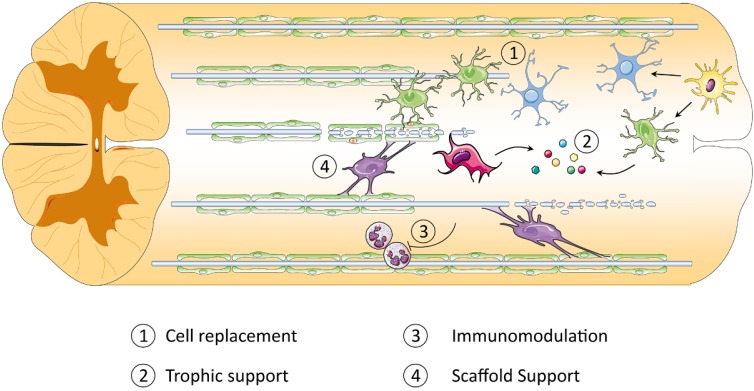
iPSC-derived cells exert their therapeutic effects for the treatment of SCI through different mechanisms. Some transplanted cells, like iPSC-derived neural progenitor cells (NPCs), can replace lost or damaged cells through differentiation or transdifferentiation into mature neurons and oligodendrocytes ①. iPSC-derived neurons and oligodendrocytes can also potentially replace lost or damaged cells. iPSC-derived cells, such as mesenchymal stromal cells (MSCs), NPCs astrocytes and oligodendrocytes, provide neurotrophic factors (like GDNF, NGF, BDNF and CNTF), which are crucial to enhancing neuronal regeneration and survival ②. iPSC-derived MSCs can also be beneficial to SCI by downregulating inhibitory molecules and immunomodulation ③. iPSC-derived astrocytes and oligodendrocytes can potentially modulate the environment and provide a scaffold support for the regeneration of axons ④.

To date, several different cell types have been successfully derived from iPSCs and have been transplanted into animal models of SCI. These studies have provided a proof of principle that iPSCs can be successfully differentiated *in vitro* to yield desirable progeny. They can be safely transplanted into models of SCI and survive, integrate and differentiate into desired phenotypes, as well as promoting functional recovery with an outcome comparable to the counterpart ESC therapy. iPSC-derived cells can be useful in the treatment of SCI through cell replacement and restoration of lost myelin and through trophic support, which results in the induction of neuroprotection and a reduction in cell loss. They may also be a source of increased regeneration and neuroplasticity. Cytokines and chemokines, which are secreted by iPSC-derived cells, can also have immunomodulatory effects. iPSC-derived cells can help remodel the physical structure of the tissue following injury to make it a less inhibitory and more permissive substrate for neural regeneration. The influence of iPSC-derived cells on astrogliosis at the early stages of injury can halt the expansion of the cystic cavity. The different potential cellular and molecular mechanisms through which iPSC-derived cells can exert their therapeutic effects in SCI are illustrated in Figure 3.

### 3.1. iPSC-Derived NPCs

One of the most promising cell types that has been studied so far for the treatment of SCI are NPCs. However, as discussed in the previous section, the availability of adult NPCs for SCI patients is limited, if even available at all. NPCs can also be derived from ESCs. However, the logistical and ethical issues surrounding the use of ESCs are quite significant. iPSCs present an alternative and potentially clinically attractive approach for the derivation of NPCs.

Several protocols have been developed to differentiate iPSCs into neural precursors and specific neuronal and glial lineages. In our lab, definitive neural progenitor cells were recently generated from piggy-Bac transposon iPSCs, and by inducing the NOTCH signaling pathway, we enhanced NPC generation with reduced expression of pluripotency and nonectodermal markers [61]. We have shown that this method is safe and effective [61]. Other studies using iPSC-NPCs in rodent and primate models of SCI have indicated that transplanted cells differentiate into neurons and glia *in vivo*, enhance remyelination and axon regeneration, supporte the survival of endogenous neurons and promote locomotor recovery and sensory responses [91,92,93]. These pre-clinical studies led to the launch of a collaborative team by Okano and Yamanaka laboratories, who are currently planning a clinical trial for hiPSC-derived NPC transplantation for SCI patients in the sub-acute phase. This study will use clinical-grade integration-free human iPSC lines that will be generated by Kyoto University’s Center for iPS Cell Research and Application (CiRA).

The Okano group has done several pioneering studies examining the transplantation of iPSC-derived NPCs for the treatment of SCI [92,94,95]. They tested different iPSC lines derived from mouse embryonic fibroblasts (MEF) or mouse tail tip fibroblasts (TTF). The iPSCs were differentiated into NPCs, and the tumorigenicity of each NPC line was pre-evaluated by transplantation into the brains of immunocompromised mice. They proposed that each iPSC line has to be pre-evaluated to assess teratoma formation after cell transplantation in animal models due to the differences among iPSC lines in differentiation capacity and teratoma formation. In this study, around 9.5 × 10^5^ cells were transplanted into the spinal cord in a T10 contusion mouse model of SCI at nine days after injury, the time window when most of the inflammatory responses are reduced. The survival rate of transplanted cells was around 20%, and they differentiated into 30% neurons, 50% astrocytes and 15% oligodendrocytes. In the transplantation group, motor function was restored for a long period of time without tumors developing. This study showed that the functional recovery after transplantation of iPSC-derived neurospheres is attributable to three possible mechanisms: (1) remyelination by mouse iPSC-derived oligodendrocytes; (2) axonal regrowth; and (3) trophic support. Those transplanted cells that differentiated into immature astrocytes may play a role in the guidance of regenerating axons [95]. The Okano group next proceeded to test the efficiency of iPSCs derived from human fibroblasts [94]. hiPSC-derived NPCs were transplanted into the spinal cord of NOD-SCID (non-obese diabetic-server combine immunodeficiency) mice with a T10 contusion injury. Transplanted cells survived, migrated and differentiated toward all neural cell fates (50% neurons, astrocytes and oligodendrocytes) and contributed to restoring motor function. These neurons could integrate into the host tissue and functioned as interneurons. They formed synapses with the host neurons and contributed to the reconstruction of neural circuits. This preclinical study serves to validate hiPSCs as a source of neural cells and represents an important step towards clinical practice [94]. In their next step towards the clinical application of hiPSC-NPCs in the treatment of SCI, they used a primate model of SCI [92]. This primate study demonstrated that hiPSC-NPCs can promote long-term functional recovery without tumorigenicity [92]. Similarly, Fujimoto *et al.* (2012) have shown that the transplantation of hiPSC-derived NPCs into a T9 contusion model of SCI in NOD-SCID mice could result in functional recovery [91]. NPCs were derived from iPSCs in a monolayer procedure, and around 10 × 10^5^ cells were transplanted into the lesion epicenter at seven days post-SCI. Grafted cells showed a survival rate of 20% and differentiated into 75% neurons, 20% astrocytes and 1% oligodendrocytes. Differentiated neurons were able to form synapses with endogenous neurons [91].

In a highly clinically-relevant study of chronic cervical SCI, Nutt *et al.* (2013) used a human iPSC line derived from human fetal lung fibroblasts for the generation of NPCs. This study transplanted 2 × 10^5^ cells into a C4 contusion rat model of SCI at the chronic time point of four weeks after injury. By four weeks after transplant, hiPSC-NPCs were mainly differentiated to astrocytes (30%) and neurons (15%), but no oligodendrocyte marker was detected. However by eight weeks after transplantation, transplanted cells with an oligodendrocyte marker (17%) were detected, though none could convincingly form myelin. Despite thorough integration and differentiation into both neurons and glia, assessment of behavioral recovery indicated that transplantation of hiPSC-NPCs did not confer any significant improvement in functional recovery. This study suggests that the best time for cell transplantation for the treatment of SCI is likely to be in the acute phases [93]. In concordance, Romanyuk *et al.*, 2014, transplanted hiPSC-NPCs into the spinal cord one week after a balloon-induced compression SCI at T8–T9. The animals were subjected to triple drug immunosuppression. Cell transplantation resulted in increased axonal regrowth, reduced lesion cavity size and improved hindlimb functional recovery, which may be due to trophic support from the cell transplant to the spared axons [96].

In a recent study by the Tuszynski group, human iPSCs derived from the dermal fibroblasts of an 89-year-old man were differentiated into NPCs [97]. These hiPSC-NPCs were used for transplantation into the spinal cord two weeks after C5 hemisection SCI in immunodeficient rats. Grafted hiPSC-NPCs showed a high survival rate three months post-transplantation and were distributed through most of the lesion. The majority of grafted hiPSC-NPCs (71%) were differentiated into neurons, and around 18% of grafted cells differentiated into astrocytes, but no oligodendrocytes were detected amongst transplanted cells. The differentiated neurons could extend their axons directly out of the lesion site and into the host spinal cord. Interestingly, these axons extended over very long distances in the host spinal cord, continuing to extend into the brain and even reaching the olfactory bulb. However, graft-derived human axons were not detectably myelinated by rat host oligodendrocytes. The synaptic structures were also formed between graft-derived human axons and host dendrites. Furthermore, host axons were shown to be capable of growing into grafted hiPSC-derived NPCs [97].

### 3.2. iPSC-Derived Oligodendrocyte Progenitor Cells

Results from our laboratory and others show that functional recovery after NPC transplantation may be chiefly attributed to remyelination of host axons by myelinating oligodendrocyte progenitors differentiated from NPCs [22,23,98,99,100,101]. Therefore, a more direct approach of transplanting oligodendrocyte progenitor cells (OPCs) straight into the spinal cord may be warranted. Several protocols have been established to generate OPCs from iPSCs [102,103], including one protocol from our group, though none of these iPSC-derived OPCs have been used in models of SCI yet. However, there has been extensive research on the application of ESC-derived OPCs for the treatment of SCI, and the application of iPSC-derived OPCs is reinforced by this. The ESC-derived OPC experiments showed that their transplantation resulted in remyelination of spared axons [24,104], improved behavioral and electrophysiological outcomes [105], restoration of forelimb motor function, improved forelimb stride length, reduced cavitation and resulted in better white and gray matter sparing [24,104]. These exciting results led to FDA approval for the world’s first phase I clinical trial by Geron Corporation for the transplantation of hESC-derived OPCs into individuals with thoracic (T3–T11) SCI on January 23, 2009. hESC-OPCs were administered into the lesion site within 14 days of injury with a low dose of two million cells. The follow-up studies on the five patients have shown no serious side effects after cell transplantation. In four of the five patients, MRI scans showed that the injury site shrank and that the cells may have had some positive effects in reducing the deterioration of spinal cord tissue. However in November, 2011, Geron announced that it had ended its SCI stem cell research program for financial reasons [106,107]. In 2013, Asterias Biotherapeutics, Inc. purchased Geron’s hESC-OPCs and recently obtained FDA approval for a dose escalation study of patients with spinal cord injury with high level (cervical) injuries [108].

### 3.3. iPSC Derived Motor Neurons

Stem cell-derived motor neurons (MNs) are increasingly utilized as cellular replacement strategies for the treatment of SCI. Motor neurons (MNs) and motor neuron progenitors (MNPs) have been successfully generated from iPSCs [109]. There are several protocols established for the generation of MNs from iPSCs. In one protocol, the exogenous expression of MN-specific factors, neurogenin 2 (NGN2), islet-1 (ISL-1) and LIM/homeobox protein 3 (LHX3), in hiPSCs derived from human fibroblasts resulted in the generation of motor neurons. There are also some other successful protocols for the generation of MNPs and MNs from iPSCs involving the sequential use of reprogramming factors, such as bFGF, activin, retinoic acid (RA) and Sonic hedgehog (SHH), in addition to growth factors, such as GDNF, BDNF and CNTF [110,111]. Although the efficiency of iPSC-derived MNs has not been tested yet for the treatment of SCI, previous data from ESC-derived MNs suggest that the application of iPSC-derived MNs may be promising. Transplantation of ESC-derived MNPs into the spinal cord of an adult rat after SCI resulted in enhanced sprouting of endogenous axons [44,45], and MNPs were shown to be able to mature into MNs and resulted in the improvement of functional recovery when transplanted *in vivo* [112].

### 3.4. iPSC-Derived Neural Crest Cells

Neural crest cells originate from cells at the border between the neuroectoderm and the surface ectoderm. They are a transient population of cells that give rise to neurons and the glial cells of the peripheral nervous system. They can be differentiated *in vitro* into Schwann cells (SC) by neuregulin-1 [113], which are capable of myelinating sensory axons *in vitro* [114], and can potentially be used for transplantation into SCI. Neural crest stem cells (NCSCs) are capable of integrating into spinal cord tissue and differentiating into neurons and myelinating oligodendrocytes [115,116]. However, human neural crest cells are difficult to obtain, because of their transient nature and the limited availability of human fetal cells [114]. There are several protocols established to derive NCSCs from iPSCs [117,118]. These iPSC-derived NCSCs can also be differentiated into Schwann cells [117,118]. hiPSC-derived NCSCs have been used *in vivo* for neural tissue engineering in athymic rat models of peripheral nerve injury. They were shown to differentiate into Schwann cells and participate in the myelination of regenerating axons [119]. The first description of iPSC-NCSC survival and integration into the spinal cord was demonstrated in a lamb spina bifida model [120], findings that support the potential application of iPSC-derived NCSCs for the treatment of SCI.

### 3.5. iPSC Derived Astrocytes

Although reactive astrocytes proliferate, form a glial scar, and secrete inhibitory agents, such as chondroitin sulfate proteoglycan, there are reports showing that the transplantation of purified astrocytes promotes axonal regeneration and functional recovery in a rat model of SCI [121]. The compaction of the lesion center and seclusion of inflammatory cells by migrating reactive astrocytes seem to underlie this beneficial effect [122,123]. Astrocytes can be derived from iPSCs [124,125,126]. In one study, astrocytes derived from iPSCs were transplanted three and seven days after T9–10 level SCI in rats. The transplanted cells survived in the spinal cords eight weeks after transplantation, but they did not result in significant locomotor recovery. However, astrocyte transplantation increased the sensitivity to mechanical stimulus and thermal hyperalgesia [125].

### 3.6. iPSC-Derived MSCs

MSCs are a promising cell source for the treatment of SCI. Although easy access to MSCs is recognized as a great advantage, extended *in vitro* culture reduces the differentiation potential of MSCs, limiting their therapeutic efficacy. Bone marrow-derived MSCs, have a limited capacity to proliferate, quickly lose differentiation potential and reduce protective factors during *ex vivo* expansion before possible therapeutic use [127]. MSCs derived from iPSCs have the potential to be expanded indefinitely without senescence [128]. To overcome the limitations of MSCs, iPSC-derived MSCs have been considered as a promising alternative for cell therapy.

MSCs have been successfully generated from iPSCs [127,129], and interestingly, they have been shown to have greater regenerative potential compared to MSCs derived from bone marrow [127]. This may be attributable to superior survival and engraftment after transplantation, because of higher telomerase activity and less senescence as compared to bone marrow-derived MSCs [127]. Future studies should examine the efficiency of iPSC-derived MSCs on different clinically relevant SCI models and compare them to umbilical cord-derived or bone marrow-derived MSCs.

## 4. Future Approaches and Prospects

### 4.1. Using Directly Reprogrammed Cells for the Treatment of SCI

The process of deriving iPSC lines and subsequently inducing differentiation is very time consuming and inefficient (0.01%–1% cell yield). In addition, the use of pluripotent-derived cells might lead to the development of tumors if not properly controlled. The transdifferentiation of one mature somatic cell into another mature somatic cell without undergoing an intermediate pluripotent state or progenitor cell type has become possible in recent years. It is now possible to directly convert fully differentiated mature cells into a variety of other cell types, while bypassing an intermediate pluripotent state. Bypassing the intermediate pluripotent state reduces the time required for generating a specific cell type and, more importantly, reduces the risk of teratoma formation. It is postulated that the future direction of cell therapy will shift from iPSCs to directly reprogrammed cells. Different cell types with potential application for the treatment of SCI have been generated recently using direct reprogramming methods [130].

#### 4.1.1. iNPC

Several studies have recently demonstrated the direct induction of neural progenitor cells (NPCs) from human and mouse fibroblasts using a range of pluripotent and neural transcription factors. Kim *et al.* (2012) have shown that fibroblasts can be directly reprogrammed into NPCs (iNPCs) by using a combination of Yamanaka factors and growth factors in culture media [130]. These iNPCs could not be maintained for more than three to five passages and lacked the potential to differentiate into oligodendrocytes. Later on, the Wernig lab overcame this problem and were able to generate self-renewing tripotent NPCs that could be differentiated not only into neurons and astrocytes, but also into oligodendrocytes [131]. In their method, they used Sox2, FoxG1 and Brn2 reprogramming factors. Removing Brn2 from this combination gave rise to the formation of bipotent iNPCs that could only differentiate into astrocytes and neurons [131]. Several other combinations of factors that can directly reprogram mouse or human fibroblast into iNPCs have subsequently been discovered [132,133,134]. Interestingly, all of these combinations had SOX2 in common. However, recently, Mitchel *et al.* (2014) showed that tripotent iNPCs can be generated from human fibroblast using only one reprogramming factor, Oct4 [135].

#### 4.1.2. iOPC

As discussed earlier, myelination by transplanted cells has a great impact on functional recovery after SCI. Myelination is mainly accomplished by oligodendrocyte progenitor cells (OPCs) [136]. However, sources of OPCs are largely restricted, and they have limited expansion capacity. Recently, Najm *et al.* (2013) have succeeded in the direct generation of OPCs (iOPCs) from mice fibroblasts by using eight reprogramming factors (Olig1, Olig2, Nkx2.2, Nkx6.2, Sox10, ST18, Myrf and Myt1), collectively referred to as 8TF (eight transcription factors). These iOPCs were capable of generating compact myelin in hypomyelinated shiverer mice [137]. More recently, the Wernig group was able to generate iOPCs from rodent fibroblasts just by using the three factors, Sox10, Olig2 and Zfp536. These iOPCs had the ability to differentiate into oligodendrocytes *in vitro* and to myelinate host axons after transplantation into the demyelinated shiverer mouse brain [138].

#### 4.1.3. iN

Transplantation of neurons and motor neurons has also shown promise as a cellular therapy in animal models of SCI. Several studies have demonstrated that combinations of neural transcription factors and/or microRNAs can directly convert both mouse and human fibroblasts into neuronal cells, including dopaminergic and motor neurons [139,140,141]. The Wernig group has recently shown that the three factors, Ascl1, Brn2 and Myt1l, are sufficient to rapidly and efficiently convert mouse embryonic and human postnatal fibroblasts into functional neurons [140,141]. In another attempt, microRNA (miR-124) and two transcription factors (Myt1l and Brn2) [142] or just Ascl1 [143] were shown to be sufficient to directly reprogram postnatal and adult human primary dermal fibroblasts into hiN (human-induced neurons). Functionally induced motor neurons (iMNs) can be generated from mouse or human fibroblasts by using seven to eight factors (Ascl1, Brn2, Myt1l, Lhx3, Hb9, Isl1, Ngn2 and NeuroD1) [144]. iMNs displayed the electrophysiological characteristics of MNs, and they formed functional synapses with muscle fibers in culture. They were also capable of extending axons into the periphery when transplanted into the developing chick spinal cord [144].

### 4.2. World’s First iPSC Clinical Trial

The start of the first ever clinical trial using human iPSCs at the RIKEN (Rikagaku Kenkyūjo) Center for Developmental Biology in Japan has raised a lot of hope for the treatment of human injury and disease, including SCI. This trial is using retinal pigment epithelium (RPE) cells derived from hiPSCs to treat age-related macular degeneration (AMD).

The Takahashi group at the RIKEN Center for Developmental Biology in Japan began a clinical pilot study to determine the safety and feasibility of using autologous hiPSC-derived RPE cell sheets in the treatment of wet AMD. The first patient was implanted with a 1.3 by 3.0-mm hiPSC-derived RPE sheet on September 12, 2014, in a 2-h procedure [145]. No initial complications from the surgery have been reported, and the patient will be followed up monthly for the first 24 weeks, bi-monthly for the next 28 weeks and then yearly for the next three years. Stem cell scientists around the world will be following this trial with interest.

## 5. Conclusions

There have been considerable advances in cell therapies for the treatment of SCI, some of which have entered clinical trials. iPSCs provide a cell source that has characteristics of embryonic stem cells, but are associated with fewer ethical and moral issues. In addition, they can be sourced from autologous sources, which may decrease the risk of immune rejection. However, there are some concerns about the use of iPSCs clinically, since many of the induction methods can increase the risk of tumors and have reprogramming efficiencies that would be too low for clinical use. In some cases, the issue of tumorigenicity may be due to partial reprogramming of the iPSCs, which may result in differentiated iPSCs reverting back to a pluripotent state [146]. Another limitation of using iPSCs clinically is that the histocompatibility of the cells may increase the risk of immune rejection. MHC I molecules make iPSCs a target of direct or indirect allorecognition [147]. Therefore, there is a need to develop histocompatible iPSC lines or to rely on patient-specific iPSCs, which adds to the time required before the treatment of the patient can begin. Lastly, there is a need to optimize the growth and expansion of iPSCs for clinical use. There is a common reliance on mouse fibroblast feeder cells to support the growth of iPSCs and the use of animal products either in the culture media or matrices used to grow the cells. This increases the risk of graft rejection [148]. One solution to this has been the use of TeSR™ media (STEMCELL Technologies, BC, Canada), which is free of animal products and does not require feeder cells to support the growth of iPSCs [149]. We also need to be able to culture the cells for use in patients at appropriate numbers, which 2D culturing systems cannot support. Microcarrier systems can be used to culture the cells in bioreactors. However, the appropriate coating, size and materials used in the microcarrier systems need to be optimized to support the growth of iPSCs, since these factors have been shown to affect cell yields of hESCs [150]. There are many methods under investigation to address these issues, including non-viral induction and direct reprogramming. With continued investigation into these methods and the start of a clinical trial using iPSCs to treat AMD, the translation to clinical use for SCI is on the horizon.

## References

[1] Sekhon L.H., Fehlings M.G. (2001). Epidemiology, demographics, and pathophysiology of acute spinal cord injury. Spine.

[2] Fehlings M.G., Tator C.H. (1999). An evidence-based review of decompressive surgery in acute spinal cord injury: Rationale, indications, and timing based on experimental and clinical studies. J. Neurosurg..

[3] Fehlings M., Singh A., Tetreault L., Kalsi-Ryan S., Nouri A. (2014). Global prevalence and incidence of traumatic spinal cord injury. Clin. Epidemiol..

[4] Baptiste D.C., Fehlings M.G. (2006). Pharmacological approaches to repair the injured spinal cord. J. Neurotrauma.

[5] Rowland J.W., Hawryluk G.W.J., Kwon B., Fehlings M.G. (2008). Current status of acute spinal cord injury pathophysiology and emerging therapies: Promise on the horizon. Neurosurg. Focus.

[6] Tator C.H., Duncan E.G., Edmonds V.E., Lapczak L.I., Andrews D.F. (1993). Changes in epidemiology of acute spinal cord injury from 1947 to 1981. Surg. Neurol..

[7] Austin J.W., Fehlings M.G. (2008). Molecular mechanisms of Fas-mediated cell death in oligodendrocytes. J. Neurotrauma.

[8] Tzekou A., Fehlings M.G. (2014). Treatment of spinal cord injury with intravenous immunoglobulin G: Preliminary evidence and future perspectives. J. Clin. Immunol..

[9] Fehlings M.G., Sekhon L.H., Tator C. (2001). The role and timing of decompression in acute spinal cord injury: What do we know? What should we do?. Spine.

[10] Tator C.H., Fehlings M.G. (1991). Review of the secondary injury theory of acute spinal cord trauma with emphasis on vascular mechanisms. J. Neurosurg..

[11] Wilson J.R., Fehlings M.G. (2011). Emerging approaches to the surgical management of acute traumatic spinal cord injury. Neurotherapeutics.

[12] Fehlings M.G., Baptiste D.C. (2005). Current status of clinical trials for acute spinal cord. Injury.

[13] Wu Y., Satkunendrarajah K., Fehlings M.G. (2014). Riluzole improves outcome following ischemia-reperfusion injury to the spinal cord by preventing delayed paraplegia. Neuroscience.

[14] Fehlings M.G., Theodore N., Harrop J., Maurais G., Kuntz C., Shaffrey C.I., Kwon B.K., Chapman J., Yee A., Tighe A. (2011). A phase I/IIa clinical trial of a recombinant RHO protein antagonist in acute spinal cord injury. J. Neurotrauma.

[15] McKerracher L., Anderson K.D. (2013). Analysis of Recruitment and Outcomes in the Phase I/IIa Cethrin Clinical Trial for Acute Spinal Cord Injury. J. Neurotrauma.

[16] Tetzlaff W., Okon E.B., Karimi-Abdolrezaee S., Hill C.E., Sparling J.S., Plemel J.R., Plunet W.T., Tsai E.C., Baptiste D., Smithson L.J. (2011). A systematic review of cellular transplantation therapies for spinal cord injury. J. Neurotrauma.

[17] Vawda R., Wilcox J., Fehlings M. (2012). Current stem cell treatments for spinal cord injury. Indian J. Orthop..

[18] Tobias C.A., Shumsky J.S., Shibata M., Tuszynski M.H., Fischer I., Tessler A., Murray M. (2003). Delayed grafting of BDNF and NT-3 producing fibroblasts into the injured spinal cord stimulates sprouting, partially rescues axotomized red nucleus neurons from loss and atrophy, and provides limited regeneration. Exp. Neurol..

[19] Alexanian A.R., Svendsen C.N., Crowe M.J., Kurpad S.N. (2011). Transplantation of human glial-restricted neural precursors into injured spinal cord promotes functional and sensory recovery without causing allodynia. Cytotherapy.

[20] Cummings B.J., Uchida N., Tamaki S.J., Salazar D.L., Hooshmand M., Summers R., Gage F.H., Anderson A.J. (2005). Human neural stem cells differentiate and promote locomotor recovery in spinal cord-injured mice. Proc. Natl. Acad. Sci. USA.

[21] Emgård M., Holmberg L., Samuelsson E.-B., Bahr B.A., Falci S., Seiger A., Sundström E. (2009). Human neural precursor cells continue to proliferate and exhibit low cell death after transplantation to the injured rat spinal cord. Brain Res..

[22] Karimi-Abdolrezaee S., Eftekharpour E., Wang J., Morshead C.M., Fehlings M.G. (2006). Delayed transplantation of adult neural precursor cells promotes remyelination and functional neurological recovery after spinal cord injury. J. Neurosci. Off. J. Soc. Neurosci..

[23] Karimi-Abdolrezaee S., Eftekharpour E., Wang J., Schut D., Fehlings M.G. (2010). Synergistic effects of transplanted adult neural stem/progenitor cells, chondroitinase, and growth factors promote functional repair and plasticity of the chronically injured spinal cord. J. Neurosci. Off. J. Soc. Neurosci..

[24] Keirstead H.S., Nistor G., Bernal G., Totoiu M., Cloutier F., Sharp K., Steward O. (2005). Human embryonic stem cell-derived oligodendrocyte progenitor cell transplants remyelinat and restore locomotion after spinal cord injury. J. Neurosci. Off. J. Soc. Neurosci..

[25] Carrade D.D., Affolter V.K., Outerbridge C.A., Watson J.L., Galuppo L.D., Buerchler S., Kumar V., Walker N.J., Borjesson D.L. (2011). Intradermal injections of equine allogeneic umbilical cord-derived mesenchymal stem cells are well tolerated and do not elicit immediate or delayed hypersensitivity reactions. Cytotherapy.

[26] Hofstetter C.P., Schwarz E.J., Hess D., Widenfalk J., el Manira A., Prockop D.J., Olson L. (2002). Marrow stromal cells form guiding strands in the injured spinal cord and promote recovery. Proc. Natl. Acad. Sci. USA.

[27] Malgieri A., Kantzari E., Patrizi M.P., Gambardella S. (2010). Bone marrow and umbilical cord blood human mesenchymal stem cells: State of the art. Int. J. Clin. Exp. Med..

[28] Mothe A.J., Bozkurt G., Catapano J., Zabojova J., Wang X., Keating A., Tator C.H. (2011). Intrathecal transplantation of stem cells by lumbar puncture for thoracic spinal cord injury in the rat. Spinal Cord.

[29] Boido M., Garbossa D., Fontanella M., Ducati A., Vercelli A. (2014). Mesenchymal stem cell transplantation reduces glial cyst and improves functional outcome after spinal cord compression. World Neurosurg..

[30] Karaoz E., Kabatas S., Duruksu G., Okcu A., Subasi C., Ay B., Musluman M., Civelek E. (2012). Reduction of lesion in injured rat spinal cord and partial functional recovery of motility after bone marrow derived mesenchymal stem cell transplantation. Turk. Neurosurg..

[31] Park W.B., Kim S.Y., Lee S.H., Kim H.-W., Park J.-S., Hyun J.K. (2010). The effect of mesenchymal stem cell transplantation on the recovery of bladder and hindlimb function after spinal cord contusion in rats. BMC Neurosci..

[32] Urdzíková L., Jendelová P., Glogarová K., Burian M., Hájek M., Syková E. (2006). Transplantation of bone marrow stem cells as well as mobilization by granulocyte-colony stimulating factor promotes recovery after spinal cord injury in rats. J. Neurotrauma.

[33] Urdzíková L.M., Růžička J., LaBagnara M., Kárová K., Kubinová S., Jiráková K., Murali R., Syková E., Jhanwar-Uniyal M., Jendelová P. (2014). Human mesenchymal stem cells modulate inflammatory cytokines after spinal cord injury in rat. Int. J. Mol. Sci..

[34] Park H.-W., Lim M.-J., Jung H., Lee S.-P., Paik K.-S., Chang M.-S. (2010). Human mesenchymal stem cell-derived Schwann cell-like cells exhibit neurotrophic effects, via distinct growth factor production, in a model of spinal cord injury. Glia.

[35] Saberi H., Moshayedi P., Aghayan H.-R., Arjmand B., Hosseini S.-K., Emami-Razavi S.-H., Rahimi-Movaghar V., Raza M., Firouzi M. (2008). Treatment of chronic thoracic spinal cord injury patients with autologous Schwann cell transplantation: An interim report on safety considerations and possible outcomes. Neurosci. Lett..

[36] Dlouhy B.J., Awe O., Rao R.C., Kirby P.A., Hitchon P.W. (2014). Autograft-derived spinal cord mass following olfactory mucosal cell transplantation in a spinal cord injury patient. J. Neurosurg. Spine.

[37] Gingras M., Beaulieu M.-M., Gagnon V., Durham H.D., Berthod F. (2008). *In vitro* study of axonal migration and myelination of motor neurons in a three-dimensional tissue-engineered model. Glia.

[38] Féron F., Perry C., Cochrane J., Licina P., Nowitzke A., Urquhart S., Geraghty T., Mackay-Sim A. (2005). Autologous olfactory ensheathing cell transplantation in human spinal cord injury. Brain J. Neurol..

[39] Mackay-Sim A., Féron F., Cochrane J., Bassingthwaighte L., Bayliss C., Davies W., Fronek P., Gray C., Kerr G., Licina P. (2008). Autologous olfactory ensheathing cell transplantation in human paraplegia: A 3-year clinical trial. Brain J. Neurol..

[40] Ogawa Y., Sawamoto K., Miyata T., Miyao S., Watanabe M., Nakamura M., Bregman B.S., Koike M., Uchiyama Y., Toyama Y. (2002). Transplantation of *in vitro*-expanded fetal neural progenitor cells results in neurogenesis and functional recovery after spinal cord contusion injury in adult rats. J. Neurosci. Res..

[41] Hatami M., Mehrjardi N.Z., Kiani S., Hemmesi K., Azizi H., Shahverdi A., Baharvand H. (2009). Human embryonic stem cell-derived neural precursor transplants in collagen scaffolds promote recovery in injured rat spinal cord. Cytotherapy.

[42] Johnson P.J., Tatara A., McCreedy D.A., Shiu A., Sakiyama-Elbert S.E. (2010). Tissue-engineered fibrin scaffolds containing neural progenitors enhance functional recovery in a subacute model of SCI. Soft Matter.

[43] Lukovic D., Valdés-Sanchez L., Sanchez-Vera I., Moreno-Manzano V., Stojkovic M., Bhattacharya S.S., Erceg S. (2014). Brief Report: Astrogliosis Promotes Functional Recovery of Completely Transected Spinal Cord Following Transplantation of hESC-Derived Oligodendrocyte and Motoneuron Progenitors: Reactive Astrocytes in Spinal Cord Injury. Stem Cells.

[44] Nógrádi A., Pajer K., Márton G. (2011). The role of embryonic motoneuron transplants to restore the lost motor function of the injured spinal cord. Ann. Anat..

[45] Rossi S.L., Nistor G., Wyatt T., Yin H.Z., Poole A.J., Weiss J.H., Gardener M.J., Dijkstra S., Fischer D.F., Keirstead H.S. (2010). Histological and functional benefit following transplantation of motor neuron progenitors to the injured rat spinal cord. PLoS One.

[46] Salewski R.P., Mitchell R.A., Shen C., Fehlings M.G. (2014). Transplantation of Neural Stem Cells Clonally Derived from Embryonic Stem Cells Promotes Recovery of the Injured Mouse Spinal Cord. Stem Cells Dev..

[47] Takahashi K., Yamanaka S. (2006). Induction of Pluripotent Stem Cells from Mouse Embryonic and Adult Fibroblast Cultures by Defined Factors. Cell.

[48] Takahashi K., Tanabe K., Ohnuki M., Narita M., Ichisaka T., Tomoda K., Yamanaka S. (2007). Induction of pluripotent stem cells from adult human fibroblasts by defined factors. Cell.

[49] Yu J., Vodyanik M.A., Smuga-Otto K., Antosiewicz-Bourget J., Frane J.L., Tian S., Nie J., Jonsdottir G.A., Ruotti V., Stewart R. (2007). Induced pluripotent stem cell lines derived from human somatic cells. Science.

[50] Meng X., Neises A., Su R.-J., Payne K.J., Ritter L., Gridley D.S., Wang J., Sheng M., William Lau K.-H., Baylink D.J. (2012). Efficient Reprogramming of Human Cord Blood CD34^+^ Cells into Induced Pluripotent Stem Cells with OCT4 and SOX2 Alone. Mol. Ther..

[51] Zhao Y., Yin X., Qin H., Zhu F., Liu H., Yang W., Zhang Q., Xiang C., Hou P., Song Z. (2008). Two supporting factors greatly improve the efficiency of human iPSC generation. Cell Stem Cell.

[52] Nakagawa M., Koyanagi M., Tanabe K., Takahashi K., Ichisaka T., Aoi T., Okita K., Mochiduki Y., Takizawa N., Yamanaka S. (2008). Generation of induced pluripotent stem cells without Myc from mouse and human fibroblasts. Nat. Biotechnol..

[53] Maekawa M., Yamaguchi K., Nakamura T., Shibukawa R., Kodanaka I., Ichisaka T., Kawamura Y., Mochizuki H., Goshima N., Yamanaka S. (2011). Direct reprogramming of somatic cells is promoted by maternal transcription factor GLIS1. Nature.

[54] Zhou W., Freed C.R. (2009). Adenoviral gene delivery can reprogram human fibroblasts to induced pluripotent stem cells. Stem Cells.

[55] Ban H., Nishishita N., Fusaki N., Tabata T., Saeki K., Shikamura M., Takada N., Inoue M., Hasegawa M., Kawamata S. (2011). Efficient generation of transgene-free human induced pluripotent stem cells (iPSCs) by temperature-sensitive Sendai virus vectors. Proc. Natl. Acad. Sci. USA.

[56] Kim D., Kim C.-H., Moon J.-I., Chung Y.-G., Chang M.-Y., Han B.-S., Ko S., Yang E., Cha K.Y., Lanza R. (2009). Generation of Human Induced Pluripotent Stem Cells by Direct Delivery of Reprogramming Proteins. Cell Stem Cell.

[57] Warren L., Manos P.D., Ahfeldt T., Loh Y.-H., Li H., Lau F., Ebina W., Mandal P.K., Smith Z.D., Meissner A. (2010). Highly Efficient Reprogramming to Pluripotency and Directed Differentiation of Human Cells with Synthetic Modified mRNA. Cell Stem Cell.

[58] Subramanyam D., Lamouille S., Judson R.L., Liu J.Y., Bucay N., Derynck R., Blelloch R. (2011). Multiple targets of miR-302 and miR-372 promote reprogramming of human fibroblasts to induced pluripotent stem cells. Nat. Biotechnol..

[59] Yu J., Hu K., Smuga-Otto K., Tian S., Stewart R., Slukvin I.I., Thomson J.A. (2009). Human Induced Pluripotent Stem Cells Free of Vector and Transgene Sequences. Science.

[60] Woltjen K., Michael I.P., Mohseni P., Desai R., Mileikovsky M., Hämäläinen R., Cowling R., Wang W., Liu P., Gertsenstein M. (2009). *PiggyBac* transposition reprograms fibroblasts to induced pluripotent stem cells. Nature.

[61] Salewski R.P., Buttigieg J., Mitchell R.A., van der Kooy D., Nagy A., Fehlings M.G. (2013). The generation of definitive neural stem cells from PiggyBac transposon-induced pluripotent stem cells can be enhanced by induction of the NOTCH signaling pathway. Stem Cells Dev..

[62] Huangfu D., Maehr R., Guo W., Eijkelenboom A., Snitow M., Chen A.E., Melton D.A. (2008). Induction of pluripotent stem cells by defined factors is greatly improved by small-molecule compounds. Nat. Biotechnol..

[63] Shi Y., Desponts C., Do J.T., Hahm H.S., Schöler H.R., Ding S. (2008). Induction of pluripotent stem cells from mouse embryonic fibroblasts by Oct4 and Klf4 with small-molecule compounds. Cell Stem Cell.

[64] Hou P., Li Y., Zhang X., Liu C., Guan J., Li H., Zhao T., Ye J., Yang W., Liu K. (2013). Pluripotent Stem Cells Induced from Mouse Somatic Cells by Small-Molecule Compounds. Science.

[65] Kim K., Doi A., Wen B., Ng K., Zhao R., Cahan P., Kim J., Aryee M.J., Ji H., Ehrlich L.I.R. (2010). Epigenetic memory in induced pluripotent stem cells. Nature.

[66] Kim K., Zhao R., Doi A., Ng K., Unternaehrer J., Cahan P., Hongguang H., Loh Y.-H., Aryee M.J., Lensch M.W. (2011). Donor cell type can influence the epigenome and differentiation potential of human induced pluripotent stem cells. Nat. Biotechnol..

[67] Laurent L.C., Ulitsky I., Slavin I., Tran H., Schork A., Morey R., Lynch C., Harness J.V., Lee S., Barrero M.J. (2011). Dynamic changes in the copy number of pluripotency and cell proliferation genes in human ESCs and iPSCs during reprogramming and time in culture. Cell Stem Cell.

[68] Taapken S.M., Nisler B.S., Newton M.A., Sampsell-Barron T.L., Leonhard K.A., McIntire E.M., Montgomery K.D. (2011). Karotypic abnormalities in human induced pluripotent stem cells and embryonic stem cells. Nat. Biotechnol..

[69] Young M.A., Larson D.E., Sun C.-W., George D.R., Ding L., Miller C.A., Lin L., Pawlik K.M., Chen K., Fan X. (2012). Background mutations in parental cells account for most of the genetic heterogeneity of induced pluripotent stem cells. Cell Stem Cell.

[70] Colman A., Dreesen O. (2009). Pluripotent Stem Cells and Disease Modeling. Cell Stem Cell.

[71] Chen J., Lin M., Foxe J.J., Pedrosa E., Hrabovsky A., Carroll R., Zheng D., Lachman H.M. (2013). Transcriptome comparison of human neurons generated using induced pluripotent stem cells derived from dental pulp and skin fibroblasts. PLoS One.

[72] Maherali N., Ahfeldt T., Rigamonti A., Utikal J., Cowan C., Hochedlinger K. (2008). A high-efficiency system for the generation and study of human induced pluripotent stem cells. Cell Stem Cell.

[73] Park I.-H., Lerou P.H., Zhao R., Huo H., Daley G.Q. (2008). Generation of human-induced pluripotent stem cells. Nat. Protoc..

[74] Yamanaka S. (2009). Elite and stochastic models for induced pluripotent stem cell generation. Nature.

[75] Aasen T., Izpisúa Belmonte J.C. (2010). Isolation and cultivation of human keratinocytes from skin or plucked hair for the generation of induced pluripotent stem cells. Nat. Protoc..

[76] Aasen T., Raya A., Barrero M.J., Garreta E., Consiglio A., Gonzalez F., Vassena R., Bilić J., Pekarik V., Tiscornia G. (2008). Efficient and rapid generation of induced pluripotent stem cells from human keratinocytes. Nat. Biotechnol..

[77] Linta L., Stockmann M., Kleinhans K.N., Böckers A., Storch A., Zaehres H., Lin Q., Barbi G., Böckers T.M., Kleger A. (2012). Rat embryonic fibroblasts improve reprogramming of human keratinocytes into induced pluripotent stem cells. Stem Cells Dev..

[78] Hu Q., Friedrich A.M., Johnson L.V., Clegg D.O. (2010). Memory in induced pluripotent stem cells: Reprogrammed human retinal-pigmented epithelial cells show tendency for spontaneous redifferentiation. Stem Cells Dayt. Ohio.

[79] Utikal J., Maherali N., Kulalert W., Hochedlinger K. (2009). Sox2 is dispensable for the reprogramming of melanocytes and melanoma cells into induced pluripotent stem cells. J. Cell Sci..

[80] Loh Y.-H., Agarwal S., Park I.-H., Urbach A., Huo H., Heffner G.C., Kim K., Miller J.D., Ng K., Daley G.Q. (2009). Generation of induced pluripotent stem cells from human blood. Blood.

[81] Brockmann F., Kramer M., Bornhäuser M., Ehninger G., Hölig K. (2013). Efficacy and side effects of granulocyte collection in healthy donors. Transfus. Med. Hemother..

[82] Ramos-Mejía V., Montes R., Bueno C., Ayllón V., Real P.J., Rodríguez R., Menendez P. (2012). Residual expression of the reprogramming factors prevents differentiation of iPSC generated from human fibroblasts and cord blood CD34^+^ progenitors. PLoS One.

[83] Giorgetti A., Montserrat N., Aasen T., Gonzalez F., Rodríguez-Pizà I., Vassena R., Raya A., Boué S., Barrero M.J., Corbella B.A. (2009). Generation of induced pluripotent stem cells from human cord blood using OCT4 and SOX2. Cell Stem Cell.

[84] Haase A., Olmer R., Schwanke K., Wunderlich S., Merkert S., Hess C., Zweigerdt R., Gruh I., Meyer J., Wagner S. (2009). Generation of induced pluripotent stem cells from human cord blood. Cell Stem Cell.

[85] Red-Horse K., Zhou Y., Genbacev O., Prakobphol A., Foulk R., McMaster M., Fisher S.J. (2004). Trophoblast differentiation during embryo implantation and formation of the maternal-fetal interface. J. Clin. Investig..

[86] Bunnell B.A., Flaat M., Gagliardi C., Patel B., Ripoll C. (2008). Adipose-derived stem cells: Isolation, expansion and differentiation. Methods San Diego Calif..

[87] Sun N., Panetta N.J., Gupta D.M., Wilson K.D., Lee A., Jia F., Hu S., Cherry A.M., Robbins R.C., Longaker M.T. (2009). Feeder-free derivation of induced pluripotent stem cells from adult human adipose stem cells. Proc. Natl. Acad. Sci. USA.

[88] Qu X., Liu T., Song K., Li X., Ge D. (2012). Induced pluripotent stem cells generated from human adipose-derived stem cells using a non-viral polycistronic plasmid in feeder-free conditions. PLoS One.

[89] Kim J.B., Greber B., Araúzo-Bravo M.J., Meyer J., Park K.I., Zaehres H., Schöler H.R. (2009). Direct reprogramming of human neural stem cells by OCT4. Nature.

[90] Kramer A.S., Harvey A.R., Plant G.W., Hodgetts S.I. (2013). Systematic review of induced pluripotent stem cell technology as a potential clinical therapy for spinal cord injury. Cell Transplant..

[91] Fujimoto Y., Abematsu M., Falk A., Tsujimura K., Sanosaka T., Juliandi B., Semi K., Namihira M., Komiya S., Smith A. (2012). Treatment of a mouse model of spinal cord injury by transplantation of human induced pluripotent stem cell-derived long-term self-renewing neuroepithelial-like stem cells. Stem Cells Dayt. Ohio.

[92] Kobayashi Y., Okada Y., Itakura G., Iwai H., Nishimura S., Yasuda A., Nori S., Hikishima K., Konomi T., Fujiyoshi K. (2012). Pre-Evaluated Safe Human iPSC-Derived Neural Stem Cells Promote Functional Recovery after Spinal Cord Injury in Common Marmoset without Tumorigenicity. PLoS One.

[93] Nutt S.E., Chang E.-A., Suhr S.T., Schlosser L.O., Mondello S.E., Moritz C.T., Cibelli J.B., Horner P.J. (2013). Caudalized human iPSC-derived neural progenitor cells produce neurons and glia but fail to restore function in an early chronic spinal cord injury model. Exp. Neurol..

[94] Nori S., Okada Y., Yasuda A., Tsuji O., Takahashi Y., Kobayashi Y., Fujiyoshi K., Koike M., Uchiyama Y., Ikeda E. (2011). Grafted human-induced pluripotent stem-cell—Derived neurospheres promote motor functional recovery after spinal cord injury in mice. Proc. Natl. Acad. Sci. USA.

[95] Tsuji O., Miura K., Okada Y., Fujiyoshi K., Mukaino M., Nagoshi N., Kitamura K., Kumagai G., Nishino M., Tomisato S. (2010). Therapeutic potential of appropriately evaluated safe-induced pluripotent stem cells for spinal cord injury. Proc. Natl. Acad. Sci. USA.

[96] Romanyuk N., Amemori T., Turnovcova K., Prochazka P., Onteniente B., Sykova E., Jendelová P. (2014). Beneficial effect of human induced pluripotent stem cell-derived neural precursors in spinal cord injury repair. Cell Transplant..

[97] Lu P., Woodruff G., Wang Y., Graham L., Hunt M., Wu D., Boehle E., Ahmad R., Poplawski G., Brock J. (2014). Long-Distance Axonal Growth from Human Induced Pluripotent Stem Cells after Spinal Cord Injury. Neuron.

[98] Hawryluk G.W.J., Mothe A.J., Chamankhah M., Wang J., Tator C., Fehlings M.G. (2012). *In vitro* characterization of trophic factor expression in neural precursor cells. Stem Cells Dev..

[99] Hawryluk G.W.J., Spano S., Chew D., Wang S., Erwin M., Chamankhah M., Forgione N., Fehlings M.G. (2014). An Examination of the Mechanisms by Which Neural Precursors Augment Recovery Following Spinal Cord Injury: A Key Role for Remyelination. Cell Transplant..

[100] Karimi-Abdolrezaee S., Schut D., Wang J., Fehlings M.G. (2012). Chondroitinase and growth factors enhance activation and oligodendrocyte differentiation of endogenous neural precursor cells after spinal cord injury. PLoS One.

[101] Yasuda A., Tsuji O., Shibata S., Nori S., Takano M., Kobayashi Y., Takahashi Y., Fujiyoshi K., Hara C.M., Miyawaki A. (2011). Significance of remyelination by neural stem/progenitor cells transplanted into the injured spinal cord. Stem Cells Dayt. Ohio.

[102] Douvaras P., Wang J., Zimmer M., Hanchuk S., O’Bara M.A., Sadiq S., Sim F.J., Goldman J., Fossati V. (2014). Efficient Generation of Myelinating Oligodendrocytes from Primary Progressive Multiple Sclerosis Patients by induced Pluripotent Stem Cells. Stem Cell Rep..

[103] Wang S., Bates J., Li X., Schanz S., Chandler-Militello D., Levine C., Maherali N., Studer L., Hochedlinger K., Windrem M. (2013). Human iPSC-Derived Oligodendrocyte Progenitor Cells Can Myelinate and Rescue a Mouse Model of Congenital Hypomyelination. Cell Stem Cell.

[104] Sharp J., Frame J., Siegenthaler M., Nistor G., Keirstead H.S. (2010). Human embryonic stem cell-derived oligodendrocyte progenitor cell transplants improve recovery after cervical spinal cord injury. Stem Cells Dayt. Ohio.

[105] Kerr C.L., Letzen B.S., Hill C.M., Agrawal G., Thakor N.V., Sterneckert J.L., Gearhart J.D., All A.H. (2010). Efficient differentiation of human embryonic stem cells into oligodendrocyte progenitors for application in a rat contusion model of spinal cord injury. Int. J. Neurosci..

[106] Bretzner F., Gilbert F., Baylis F., Brownstone R.M. (2011). Target Populations for First-In-Human Embryonic Stem Cell Research in Spinal Cord Injury. Cell Stem Cell.

[107] Wilcox J.T., Cadotte D., Fehlings M.G. (2012). Spinal cord clinical trials and the role for bioengineering. Neurosci. Lett..

[108] Treatment for Spinal Cord Injury to Start Clinical Trial Funded by California’s Stem Cell Agency. http://www.cirm.ca.gov/about-cirm/newsroom/press-releases/08262014/treatment-spinal-cord-injury-start-clinical-trial-funded.

[109] Sareen D., O’Rourke J.G., Meera P., Muhammad A.K.M.G., Grant S., Simpkinson M., Bell S., Carmona S., Ornelas L., Sahabian A. (2013). Targeting RNA Foci in iPSC-Derived Motor Neurons from ALS Patients with a C9ORF72 Repeat Expansion. Sci. Transl. Med..

[110] Jha B.S., Rao M., Malik N. (2014). Motor Neuron Differentiation from Pluripotent Stem Cells and Other Intermediate Proliferative Precursors that can be Discriminated by Lineage Specific Reporters. Stem Cell Rev..

[111] Karumbayaram S., Novitch B.G., Patterson M., Umbach J.A., Richter L., Lindgren A., Conway A.E., Clark A.T., Goldman S.A., Plath K. (2009). Directed differentiation of human-induced pluripotent stem cells generates active motor neurons. Stem Cells Dayt. Ohio.

[112] Erceg S., Ronaghi M., Oria M., Roselló M.G., Aragó M.A.P., Lopez M.G., Radojevic I., Moreno-Manzano V., Rodríguez-Jiménez F.-J., Bhattacharya S.S. (2010). Transplanted oligodendrocytes and motoneuron progenitors generated from human embryonic stem cells promote locomotor recovery after spinal cord transection. Stem Cells Dayt. Ohio.

[113] Sieber-Blum M., Grim M., Hu Y.F., Szeder V. (2004). Pluripotent neural crest stem cells in the adult hair follicle. Dev. Dyn..

[114] Liu Q., Spusta S.C., Mi R., Lassiter R.N.T., Stark M.R., Höke A., Rao M.S., Zeng X. (2012). Human neural crest stem cells derived from human ESCs and induced pluripotent stem cells: Induction, maintenance, and differentiation into functional Schwann cells. Stem Cells Transl. Med..

[115] Sieber-Blum M., Schnell L., Grim M., Hu Y.F., Schneider R., Schwab M.E. (2006). Characterization of epidermal neural crest stem cell (EPI-NCSC) grafts in the lesioned spinal cord. Mol. Cell. Neurosci..

[116] Trolle C., Konig N., Abrahamsson N., Vasylovska S., Kozlova E.N. (2014). Boundary cap neural crest stem cells homotopically implanted to the injured dorsal root transitional zone give rise to different types of neurons and glia in adult rodents. BMC Neurosci..

[117] Kreitzer F.R., Salomonis N., Sheehan A., Huang M., Park J.S., Spindler M.J., Lizarraga P., Weiss W.A., So P.-L., Conklin B.R. (2013). A robust method to derive functional neural crest cells from human pluripotent stem cells. Am. J. Stem Cells.

[118] Lee G., Chambers S.M., Tomishima M.J., Studer L. (2010). Derivation of neural crest cells from human pluripotent stem cells. Nat. Protoc..

[119] Wang A., Tang Z., Park I.-H., Zhu Y., Patel S., Daley G.Q., Song L. (2011). Induced Pluripotent Stem Cells for Neural Tissue Engineering. Biomaterials.

[120] Saadai P., Wang A., Nout Y.S., Downing T.L., Lofberg K., Beattie M.S., Bresnahan J.C., Li S., Farmer D.L. (2013). Human induced pluripotent stem cell-derived neural crest stem cells integrate into the injured spinal cord in the fetal lamb model of myelomeningocele. J. Pediatr. Surg..

[121] Davies J.E., Huang C., Proschel C., Noble M., Mayer-Proschel M., Davies S.J.A. (2006). Astrocytes derived from glial-restricted precursors promote spinal cord repair. J. Biol..

[122] Faulkner J.R., Herrmann J.E., Woo M.J., Tansey K.E., Doan N.B., Sofroniew M.V. (2004). Reactive astrocytes protect tissue and preserve function after spinal cord injury. J. Neurosci..

[123] Renault-Mihara F., Okada S., Shibata S., Nakamura M., Toyama Y., Okano H. (2008). Spinal cord injury: Emerging beneficial role of reactive astrocytes’ migration. Int. J. Biochem. Cell Biol..

[124] Emdad L., D’Souza S.L., Kothari H.P., Qadeer Z.A., Germano I.M. (2011). Efficient Differentiation of Human Embryonic and Induced Pluripotent Stem Cells into Functional Astrocytes. Stem Cells Dev..

[125] Hayashi K., Hashimoto M., Koda M., Naito A.T., Murata A., Okawa A., Takahashi K., Yamazaki M. (2011). Increase of sensitivity to mechanical stimulus after transplantation of murine induced pluripotent stem cell-derived astrocytes in a rat spinal cord injury model. J. Neurosurg. Spine.

[126] Juopperi T.A., Kim W.R., Chiang C.-H., Yu H., Margolis R.L., Ross C.A., Ming G., Song H. (2012). Astrocytes generated from patient induced pluripotent stem cells recapitulate features of Huntington’s disease patient cells. Mol. Brain.

[127] Lian Q., Zhang Y., Zhang J., Zhang H.K., Wu X., Zhang Y., Lam F.F.-Y., Kang S., Xia J.C., Lai W.-H. (2010). Functional mesenchymal stem cells derived from human induced pluripotent stem cells attenuate limb ischemia in mice. Circulation.

[128] Jung Y., Bauer G., Nolta J.A. (2012). Concise review: Induced pluripotent stem cell-derived mesenchymal stem cells: Progress toward safe clinical products. Stem Cells.

[129] Himeno T., Kamiya H., Naruse K., Cheng Z., Ito S., Kondo M., Okawa T., Fujiya A., Kato J., Suzuki H. (2013). Mesenchymal stem cell-like cells derived from mouse induced pluripotent stem cells ameliorate diabetic polyneuropathy in mice. BioMed. Res. Int..

[130] Kim J., Efe J.A., Zhu S., Talantova M., Yuan X., Wang S., Lipton S.A., Zhang K., Ding S. (2011). Direct reprogramming of mouse fibroblasts to neural progenitors. Proc. Natl. Acad. Sci. USA.

[131] Lujan E., Chanda S., Ahlenius H., Südhof T.C., Wernig M. (2012). Direct conversion of mouse fibroblasts to self-renewing, tripotent neural precursor cells. Proc. Natl. Acad. Sci. USA.

[132] Han D.W., Tapia N., Hermann A., Hemmer K., Höing S., Araúzo-Bravo M.J., Zaehres H., Wu G., Frank S., Moritz S. (2012). Direct reprogramming of fibroblasts into neural stem cells by defined factors. Cell Stem Cell.

[133] Ring K.L., Tong L.M., Balestra M.E., Javier R., Andrews-Zwilling Y., Li G., Walker D., Zhang W.R., Kreitzer A.C., Huang Y. (2012). Direct reprogramming of mouse and human fibroblasts into multipotent neural stem cells with a single factor. Cell Stem Cell.

[134] Zou Q., Yan Q., Zhong J., Wang K., Sun H., Yi X., Lai L. (2014). Direct conversion of human fibroblasts into neuronal restricted progenitors. J. Biol. Chem..

[135] Mitchell R.R., Szabo E., Benoit Y.D., Case D.T., Mechael R., Alamilla J., Lee J.H., Fiebig-Comyn A., Gillespie D.C., Bhatia M. (2014). Activation of Neural Cell Fate Programs toward Direct Conversion of Adult Human Fibroblasts into Tri-Potent Neural Progenitors Using OCT-4. Stem Cells Dev..

[136] Franklin R.J.M., Ffrench-Constant C. (2008). Remyelination in the CNS: From biology to therapy. Nat. Rev. Neurosci..

[137] Najm F.J., Lager A.M., Zaremba A., Wyatt K., Caprariello A.V., Factor D.C., Karl R.T., Maeda T., Miller R.H., Tesar P.J. (2013). Transcription factor-mediated reprogramming of fibroblasts to expandable, myelinogenic oligodendrocyte progenitor cells. Nat. Biotechnol..

[138] Yang N., Zuchero J.B., Ahlenius H., Marro S., Ng Y.H., Vierbuchen T., Hawkins J.S., Geissler R., Barres B.A., Wernig M. (2013). Generation of oligodendroglial cells by direct lineage conversion. Nat. Biotechnol..

[139] Caiazzo M., Dell’Anno M.T., Dvoretskova E., Lazarevic D., Taverna S., Leo D., Sotnikova T.D., Menegon A., Roncaglia P., Colciago G. (2011). Direct generation of functional dopaminergic neurons from mouse and human fibroblasts. Nature.

[140] Pang Z.P., Yang N., Vierbuchen T., Ostermeier A., Fuentes D.R., Yang T.Q., Citri A., Sebastiano V., Marro S., Südhof T.C. (2011). Induction of human neuronal cells by defined transcription factors. Nature.

[141] Vierbuchen T., Ostermeier A., Pang Z.P., Kokubu Y., Südhof T.C., Wernig M. (2010). Direct conversion of fibroblasts to functional neurons by defined factors. Nature.

[142] Ambasudhan R., Talantova M., Coleman R., Yuan X., Zhu S., Lipton S.A., Ding S. (2011). Direct Reprogramming of Adult Human Fibroblasts to Functional Neurons under Defined Conditions. Cell Stem Cell.

[143] Chanda S., Ang C.E., Davila J., Pak C., Mall M., Lee Q.Y., Ahlenius H., Jung S.W., Südhof T.C., Wernig M. (2014). Generation of Induced Neuronal Cells by the Single Reprogramming Factor ASCL1. Stem Cell Rep..

[144] Son E.Y., Ichida J.K., Wainger B.J., Toma J.S., Rafuse V.F., Woolf C.J., Eggan K. (2011). Conversion of mouse and human fibroblasts into functional spinal motor neurons. Cell Stem Cell.

[145] Cyranoski D. Japanese woman is first recipient of next-generation stem cells. http://www.nature.com/news/japanese-woman-is-first-recipient-of-next-generation-stem-cells1.15915.

[146] Chen J., Liu H., Liu J., Qi J., Wei B., Yang J., Liang H., Chen Y., Chen J., Wu Y. (2013). H3K9 methylation is a barrier during somatic cell reprogramming into iPSCs. Nat. Genet..

[147] Scaron T., Frenzel L.P., Hescheler J. (2008). Immunological Barriers to Embryonic Stem Cell-Derived Therapies. Cells Tissues Organs.

[148] Ludwig T.E., Levenstein M.E., Jones J.M., Berggren W.T., Mitchen E.R., Frane J.L., Crandall L.J., Daigh C.A., Conard K.R., Piekarczyk M.S. (2006). Derivation of human embryonic stem cells in defined conditions. Nat. Biotechnol..

[149] Chen A.K.-L., Reuveny S., Oh S.K.W. (2013). Application of human mesenchymal and pluripotent stem cell microcarrier cultures in cellular therapy: Achievements and future direction. Biotechnol. Adv..

[150] Chen A.K.-L., Chen X., Choo A.B.H., Reuveny S., Oh S.K.W. (2011). Critical microcarrier properties affecting the expansion of undifferentiated human embryonic stem cells. Stem Cell Res..

